# Enhancing Antibiotic Effect by Photodynamic: The Case of *Klebsiella pneumoniae*

**DOI:** 10.3390/antibiotics14080766

**Published:** 2025-07-29

**Authors:** Koteswara Rao Yerra, Vanderlei S. Bagnato

**Affiliations:** 1 Department of Biomedical Engineering, College of Engineering, Texas A&M University, College Station, TX 77843, USA; 2 São Carlos Institute of Physics, University of São Paulo, São Carlos 400, SP, Brazil

**Keywords:** *Klebsiella pneumoniae*, photosensitizers, photodynamic therapy, combination of PDT and antibiotics, synergetic effect

## Abstract

**Background:** The effect of antibiotics can be severely affected by external factors. Combining the oxidative impact of photodynamic therapy with antibiotics is largely unexplored, which may result in positive results with great impact on clinical applications. In particular, that can be relevant in the case of antibiotic resistance. **Objectives:** In this study, we examined the effects of aPDT using the photosensitizers (PSs), methylene blue (MB) or Photodithazine (PDZ), both alone and in combination with the antibiotics ciprofloxacin (CIP), gentamicin (GEN), and ceftriaxone (CEF), against the Gram-negative bacterium *Klebsiella pneumoniae*. **Methods:** A standard suspension of *K. pneumoniae* was subjected to PDT with varying doses of MB and PDZ solutions, using a 75 mW/cm^2^ LED emitting at 660 nm with an energy of 15 J/cm^2^. The MICs of CIP, GEN, and CEF were determined using the broth dilution method. We also tested the photosensitizers MB or PDZ as potentiating agents for synergistic combinations with antibiotics CIP, GEN, and CEF against *K. pneumoniae*. **Results:** The results showed that MB was more effective in inhibiting survival and killing *K. pneumoniae* compared to PDZ. The tested antibiotics CIP, GEN, and CEF suppressed bacterial growth (as shown by reduced MIC values) and effectively killed *K. pneumoniae* (reduced Log CFU/mL). While antibiotic treatment or aPDT alone showed a moderate effect (1 Log_10_ to 2 Log_10_ CFU reduction) on killing *K. pneumoniae*, the combination therapy significantly increased bacterial death, resulting in a ≥3 Log_10_ to 6 Log_10_ CFU reduction. **Conclusions:** Our study indicates that pre-treating bacteria with PDT makes them more susceptible to antibiotics and could serve as an alternative for treating local infections caused by resistant bacteria or even reduce the required antibiotic dosage. This work explores numerous possible combinations of PDT and antibiotics, emphasizing their interdependence in controlling infections and the unique properties each PS-antibiotic combination offers. Clinical application for the combination is a promising reality since both are individually already adopted in clinical use.

## 1. Introduction

*Klebsiella pneumoniae* stands out as a major driver of healthcare-associated infections in hospitals worldwide, especially in cases of hospital-acquired pneumonia (HAP) or ventilator-associated pneumonia (VAP) [1]. These infections are linked to high mortality rates, and their treatment is difficult due to multidrug resistance to current therapies [1]. Antibiotics are the most commonly used treatment for bacterial infections. They work by controlling bacterial growth either through disrupting cell integrity or inhibiting essential processes like cell wall, protein, and nucleic acid synthesis [2]. The rise in antimicrobial resistance (AR) has occurred because of the widespread and sometimes improper use of antibiotics, making many bacteria resistant to treatment [2]. Key mechanisms bacteria use to develop AR include reducing drug uptake, increasing drug efflux, sequestering incoming drugs, and modifying drug targets [2]. Additionally, bacterial pathogens can form biofilms—groups of bacteria that stick together and produce an extracellular matrix—which can block antibiotics and worsen bacterial AR [2].

An alternative method for treating multidrug-resistant (MDR) bacterial infections is antimicrobial photodynamic therapy (PDT) [3]. PDT has become a promising approach for combating drug-resistant bacterial infections by avoiding the overuse of antibiotics [3,4]. It generates cytotoxic free radicals through photochemical reactions with visible light, a non-toxic dye (photosensitizer, PS), and oxygen or biological molecules [5]. PSs belong to various chemical classes, such as phenothiaziniums (e.g., methylene blue, toluidine blue, and rose bengal), tetra-pyrroles (e.g., porphyrins and their derivatives, Ce6), natural PSs (e.g., curcumin), nanostructures (e.g., C60 fullerene), tetracycline, pheophorbide (e.g., Pheo A) [5]. During PDT, PSs are excited by light of a specific wavelength in the presence of oxygen, leading to ROS production. These ROS kill bacteria by various methods, including damaging their cell wall membranes, disrupting cell survival proteins, and activating pro-cell-death factors, etc. [5]. PDT presents several limitations, including its restriction to areas that are accessible to light and its inherently weak penetration ability. Additionally, it may struggle with target specificity and exhibit reduced efficacy in hypoxic environments, and a short lifespan of ROS. However, numerous studies are currently underway to address these challenges and enhance the overall performance of PDT [3,5].

Antibiotics and PDT are two vital and promising approaches for combating bacterial infections. As antibiotic resistance continues to increase and the discovery of new antibiotics slows down, it is crucial to make the best use of existing antibiotics to effectively tackle infections [2]. Integrating PDT with antibiotics can create a constructive effect that enhances the antibacterial effectiveness of both treatments. This approach not only improves the overall efficacy against infections but also holds promise for minimizing the unwanted side effects often associated with traditional antimicrobial therapies [6]. Furthermore, unlike antibiotics, which can take hours to days to show their effect, PDT can kill bacteria in seconds to minutes through oxidative stress triggered by light [5,6]. Incorporating PDT into routine antibiotic therapy is essential, as it significantly accelerates the eradication process of bacterial infections. Antibiotic-resistant bacteria, such as *K. pneumoniae*, pose a significant challenge in treating infections. In response to this growing concern, our study introduces a novel strategy aimed at effectively eliminating infections caused by these resistant pathogens. Thus, the aim of this investigation is a comparative evaluation of the combined effect of aPDT using the photosensitizers methylene blue (MB) and Photodithazine (PDZ), both alone and in combination with antibiotics such as Ciprofloxacin (CIP), Gentamicin (GEN), and Ceftriaxone (CEF), against the Gram-negative bacterium *Klebsiella pneumoniae*. The used photosensitizers, MB and PDZ are excited by the same wavelength of light at 660 nm, and that is very convenient for the comparison that we want to make in this report. This strategy can allow for the administration of lower concentrations of each PS, thereby reducing potential side effects such as dark toxicity.

## 2. Results

### 2.1. Effect of PDT Alone on Inhibition of Klebsiella pneumoniae

**Figure 1:**
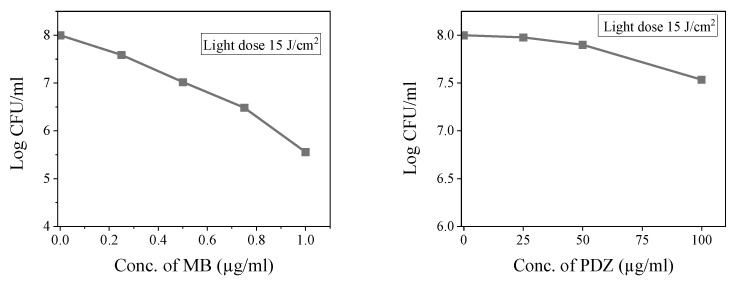
Evaluation of the photodynamic activity of photosensitizers (MB and PDZ) on *K. pneumoniae* irradiated with red LED light (660 nm). Varying the photosensitizer concentration of MB (0, 0.25, 0.5, 0.75, and 1 µg/mL), or PDZ (0, 25, 50, and 100 µg/mL). All assays used a light dose of 15 J/cm^2^. Calculated using mean and standard deviation, statistical significance was compared between each treated group (PS + PDT) to the “only PS” control group for each concentration level.

The first measurement to be performed as a reference is the individual effects of the PDT as well as the antibiotic. That will be the reference basis for the combination. To assess the influence of MB or PDZ concentration on the viability of *K. pneumoniae* ATCC 13883, bacterial cells were treated with MB or PDZ with varied concentrations of MB (0.25, 0.5, 0.75, and 1 μg/mL), or PDZ (25, 50, and 100 μg/mL), and incubated for 20 min under dark conditions, followed by irradiation with 15 J/cm^2^ using a 660 nm biotable. The assessment of aPDT’s impact on the viability of *K. pneumoniae* was conducted by measuring the colony-forming units (CFU)/mL of bacterial control cells, as well as those treated with LED (without MB or PDZ) or with 1 μg/mL MB or 100 μg/mL PDZ. The results indicated that there was no statistically significant reduction (*p* > 0.05) in the cell viability of *K. pneumoniae* across all conditions tested. However, exposing log-phase *K. pneumoniae* cultures to photosensitizers, MB or PDZ, and light reduced their viability, and observed that the effect was dependent on PS concentration (Figure 1). The results showed that upon irradiation with 15 J/cm^2^ of 660 nm light, MB was a superior survival rate inhibitor of *K. pneumoniae* as compared with PDZ (Figure 1). Compared with the control, the CFU of *K. pneumoniae* decreased by 0.42, 1.0, 1.52, and 2.45 (logarithmic scale) following treatment with 0.25, 0.5, 0.75 µg/mL, and 1.0 µg/mL of MB for 20 min, respectively. The replacement of MB by PDZ led to different results, as shown in Figure 1. There was no significant difference in CFU for the *K. pneumoniae* in the 25, 50  µg/mL of PDZ treatment groups. As the concentration of PDZ was increased to 100 μg/mL, we observed a moderate reduction in the viable count following irradiation, with a decrease of 0.47 on a logarithmic scale. We further investigated the association between the dosage of light energy and the survival of *K. pneumoniae*. The results indicated that the light dose increased from 15 J/cm^2^ to 30 J/cm^2^, significantly reducing the bacterial survival of *K. pneumoniae*, indicating that the light energy doses had a considerable effect on the growth of the tested bacteria.

### 2.2. Minimum Inhibitory Concentrations (MIC) of Antibiotics Against K. pneumoniae

We determined the MICs of the antibiotics ciprofloxacin (CIP), gentamicin (GEN), and ceftriaxone (CEF) against *K. pneumoniae*. The selected antibiotics are commonly utilized in hospital settings, with each one representing a distinct class and mechanism of action effective against bacterial infections. The CIP belongs to the fluoroquinolone class and works by inhibiting bacterial nucleic acid synthesis. The GEN, classified as an aminoglycoside, functions by stopping bacterial protein synthesis. The CEF is part of the β-lactam cephalosporin class and inhibits bacterial cell wall synthesis. The MIC assay was conducted using a serial dilution method in 96-well microplates. The results are shown in Table 1.

The MIC values of CIP, GEN, and CEF are 0.1875, 1.5 μg/mL, and 2.125 µg/mL, respectively, demonstrating that CIP is the most efficient inhibitor/concentration used, while CEF seems to be the least efficient.

**Figure 2:**
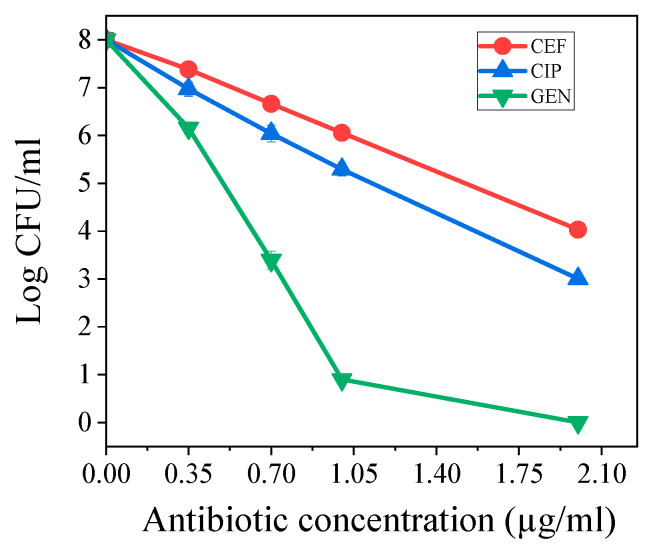
The combined effect of antibiotics (CEF, GEN, and CEF) and Photodynamic Therapy (PDT) on the viability of *K. pneumoniae*. The graph shows the count of colony-forming units (CFU/mL) as a function of tested antibiotics concentration (µg/mL) at a constant light dose of 15 J/cm^2^. The profile for GEN, indicates that it reaches full elimination much faster than the last point indicated.

The time-killing assays of antibiotics CIP, GEN, and CEF against *K. pneumoniae* are shown in Figure 2. We calculated the percentage decrease and logarithmic reduction in the *K. pneumoniae* population at each time point. This shows how the population changed, either decreasing or growing, compared to the initial inoculum. All quantitative data are subjected to statistical analysis, and the results showed that the comparison with the negative control was significant at *p* < 0.05. A sharp decrease in *K. pneumoniae* survival fraction was observed with an increase in the GEN concentration (Figure 2). The tested antibiotics at a maximal tested concentration of 2 µg/mL, Gentamicin (GEN), eliminated almost all *K. pneumoniae* within an 18 h exposure period. In comparison, ciprofloxacin (CIP) reduced the bacterial count by approximately 4.8 Log10, while ceftriaxone (CEF) achieved a reduction of around 4 Log10. These results suggested that GEN exhibited more potent bactericidal activity against *K. pneumoniae* compared to CIP and GEN in vitro. This demonstrates that the tested antibiotics CIP, GEN, and CEF can all suppress bacterial growth (as evidenced by reduced MIC values) and effectively kill the *K. pneumoniae* (reduced Log CFU/mL).

### 2.3. Effect of the PDT and Antibiotics Combination on K. pneumoniae

**Figure 3:**
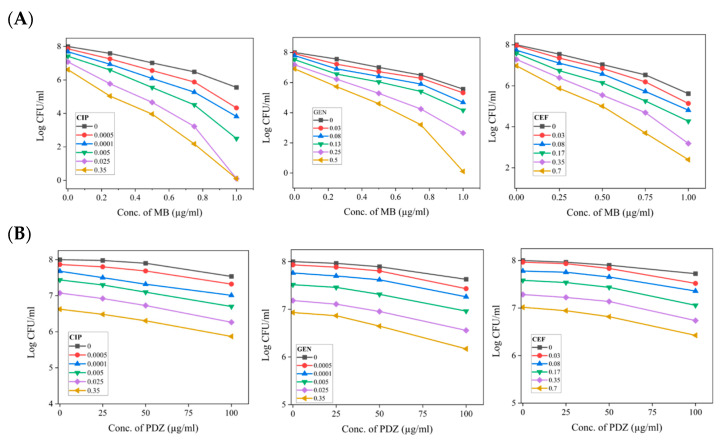
The combined effect of photosensitizers (MB or PDZ) and antibiotics (CEF, GEN, and CEF) on the viability of *K. pneumoniae*. The graph shows colony-forming units (CFU/mL) as a function of photosensitizer and antibiotic concentrations (µg/mL) at an energy dose of 15 J/cm^2^. Statistical significance was determined by comparing treated groups (PS + antibiotics) to the “only PS” control group for each concentration. Error bars represent standard deviation across replicates. (**A**) Sequence of different experiments using the three selected antibiotics as the concentration of Methylene Blue is varied. (**B**) Sequence of different experiments using the three selected antibiotics as the concentration of PDZ is varied.

We also screened the photosensitizers MB or PDZ as a potentiating agent for synergistic combinations with antibiotics CIP, GEN, and CEF against *K. pneumoniae*. We inquired whether these combinations exhibit additive effects or synergistic interactions. Synergistic interactions occur when the growth of *K. pneumoniae* is inhibited to a greater extent than would be expected from the individual effects of each component. The FIC_I_ results showed that all the tested combinations have a synergistic effect. The experiments were carried out by performing the PDT at a single selected light dose of 15 J/cm^2^, and varying doses of photosensitizers and antibiotics. At the individual capacity, the concentration, both PSs (MB or PDZ) and the antibiotics tested (CIP, GEN, and CEF) showed mild killing (0.5 to 2 Log10) of *K. pneumoniae* after 18 h (Figure 1 and Figure 2). However, combinations of MB with relatively lower concentrations of antibiotics (as compared with their effect alone) were found to be synergistic and achieved 2 to 6 log10 orders of bacteria killing (Figure 3A). Checkerboard analysis revealed that the FIC_I_ values obtained were equal to or greater than 0.5. This finding indicates the presence of synergistic interactions when combining PDT using PSs such as MB or PDZ with antibiotics, including CIP, GEN, or CEF. Particularly, as shown in Figure 3A, MB displayed potent synergy with the tested antibiotics, comprising bacterial nucleic acid synthesis inhibitors (MB and CIP), bacterial protein synthesis inhibitors (MB and GEN), and bacterial cell wall synthesis inhibitors (MB and CEF). It is interesting to note that after continuous treatment for 18 h, the combination of MB (1 µg/mL) and CIP (0.025 µg/mL and 0.35 µg/mL) completely eradicated the *K. pneumoniae* (Figure 3A). After 18 h of treatment with the MB (1 µg/mL) and GEN (0.25 µg/mL), and MB (1 µg/mL) and CEF (0.35 µg/mL) combinations, the bacterial counts of *K. pneumoniae* decreased by 5.16 and 5.21 log values, respectively, compared to the starting bacterial load (Figure 3A). The overall bactericidal efficacy order of MB and antibiotics against *K. pneumoniae* is CIP > GEN > CEF (Figure 3). The results demonstrate that the combination treatment was more effective in eliminating *K. pneumoniae*, allowing for the use of reduced antibiotic concentrations and lower doses of PSs, as illustrated in Figure 3. During this study, PDZ displayed only a moderate effect at relatively higher concentrations of 100 µg/mL (Figure 3B).

**Figure 4:**
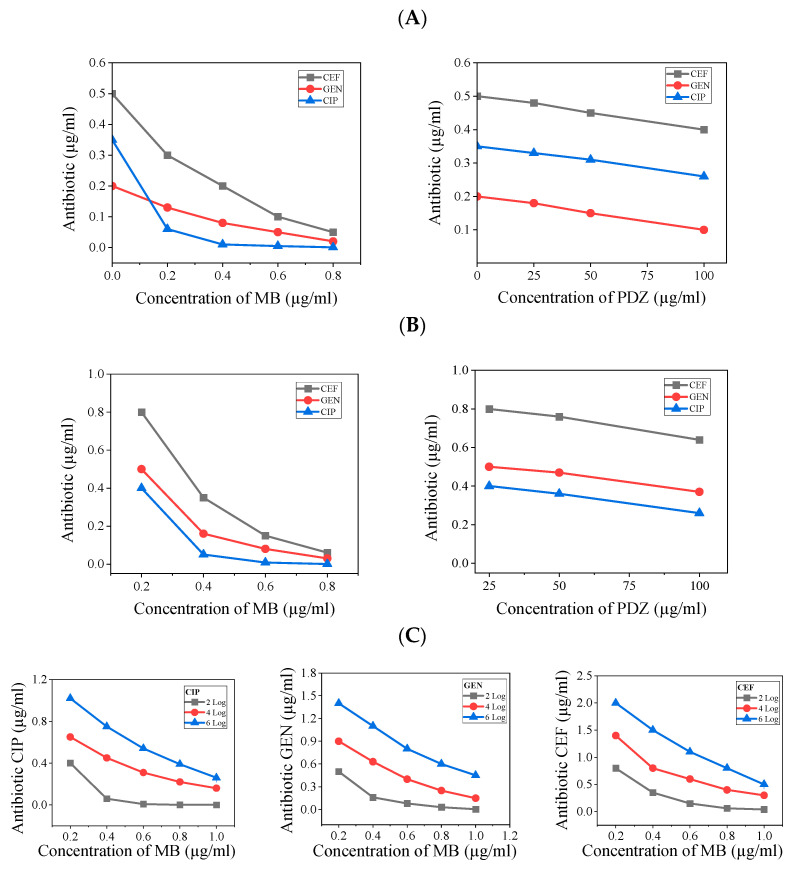
Equivalence plot between PDT under the described conditions and the concentration of antibiotics. Combination effect of Photosensitizers (MB or PDZ) and antibiotics (CEF, GEN, and CEF) against *K. pneumoniae*. (**A**) for 1 log reduction, (**B**) for 2 log reduction, (**C**) for 2, 4, 6 log reduction in each antibiotic. As the photosensitizer concentration increases, less gain is obtained, indicating a saturation tendency for high concentrations of photosensitizer.

From Figure 3, one observes that there is always more elimination with higher concentrations of antibiotic and concentration of photosensitizer, which is somewhat expected. After obtaining such results, an analysis related to the level of inhibitory action of both agents together was obtained from the presented results in Figure 4A–C. From that, the combination of antibiotic and PDT to reach a specified level of inhibition is now depicted. Such plots show the compromise between the two agents for controlling the quantity of microorganisms. The results as presented in Figure 4, allow us to understand the regimes of concentrations of both agents where strong or weak mutual dependence takes place.

To elaborate on the synergistic effect, there exists an equivalence between the effects of antibiotics and the effects of photosensitizers. To effectively determine the quantity of *K. pneumoniae* that needs to be eliminated, it is crucial to establish this number beforehand. By performing so, it becomes possible to calculate the necessary combined dosages of both the antibiotic and photosensitizer that will help achieve the intended results. This coordinated approach ensures that the treatment is both effective and precise in targeting *K. pneumoniae*. We noted that in the plot of antibiotic concentration versus photosensitizer concentration, to achieve a certain level of killing, if the curve runs parallel to the photosensitizer axis, it indicates that the combination is insensitive to PDT. Conversely, if the curve runs parallel to the antibiotic concentration axis, it suggests the combination is insensitive to the antibiotic. These are cases of no synergistic effect. However, by approaching more from one behavior or the other, we can analyze the synergistic effect. In that regard, PDZ only showed partial synergies with the tested antibiotics against *K. pneumoniae* (Figure 4). These results suggest that PDZ, in combination with the tested antibiotics, produced a weak killing effect, enhanced by PDT. Meanwhile, MB and the antibiotics seem to be in a transition, with the antibiotics being more effective at lower photosensitizer concentrations and the effect of PDT becoming more dominant as that concentration increases, reaching a peak at a certain point. Due to the more pronounced effect of MB compared to PDZ, we consider the combination of MB with the selected antibiotics over a broader range of killing levels, as shown in Figure 4. In Figure 4, when one of the plots for a specific level of inhibition draws parallel to one of the axes, that is the regime where the process becomes independent of that specific variable of concentration.

## 3. Discussion

The application of PSs combined with light for PDT presents numerous benefits. This approach effectively targets not only Gram-positive and Gram-negative bacteria but also extends its efficacy to protozoa, viruses, and fungi [5]. In comparison, traditional antibiotics typically possess a limited spectrum of antibacterial activity [7]. Additionally, PSs do not induce resistance in bacteria and are active against planktonic antibiotic-sensitive bacteria, resistant strains, and biofilm-forming bacterial species. The two PSs used in this study, MB or PDZ, were chosen because they are chemically quite different. Our results suggest that MB is a more effective inhibitor of *K. pneumoniae* compared to PDZ (Figure 1). The molecule MB contains a central heterocyclic aromatic thiazide ring system that enables a delocalized positive charge at neutral pH. It is a commercially available, inexpensive material with high purity and a stable composition. MB has a high reduction potential and a suitable hydrophilic/lipophilic balance, which allows it to cross cell membranes and facilitates intracellular absorption [8]. Chlorins are second-generation photosensitizers derived from reduced porphyrin rings. Photodithazine^®^ (PDZ), an e6 chlorine from the cyanobacterium *Spirulina platensis*, has shown promising results in aPDT [9]. When comparing the photodynamic profile of *K. pneumoniae* inactivation with MB or PDZ and their singlet oxygen (^1^O_2_) production, it was surprising to find that PDZ, which had the highest capacity for singlet oxygen generation, was the least effective in photoinactivating *K. pneumoniae*. The limited interaction of PDZ with the bacterial membrane may account for its reduced effectiveness. The primary targets of aPDT are the proteins and lipids found in the cytoplasmic membrane and the bacterial cell wall. If the photosensitizer is less effective in interacting or binding to these external structures, the overall efficiency of bacterial inactivation can be significantly diminished [9]. Furthermore, PDZ is a Ce6 derivative that has been modified with N-methyl-D-glucosamine, a solubilizing and stabilizing agent, and it has a high potential for inactivating Gram-positive bacteria [10]. The varying structures of the outer cell envelopes in Gram-positive and Gram-negative bacteria play a significant role in the effectiveness of photosensitizer penetration [11]. In Gram-negative bacteria, the outer membrane acts as a major barrier to the entry of PSs. This membrane is made up of lipopolysaccharides, which are hydrophobic and create a protective layer that limits access for hydrophilic compounds. It also has porin proteins that allow only small, hydrophilic molecules to pass through, which further blocks larger or hydrophobic compounds. Additionally, the highly negative surface charge of Gram-negative bacteria repels many PSs [11]. Our study found that the photosensitizer MB is more effective than PDZ in killing the Gram-negative bacteria *K. pneumoniae* (Figure 1).

The direct activity of all tested antibiotics on planktonic cells of *K. pneumoniae* showed that CIP was the most effective, followed by GEN, and then CEF (Table 1). The recorded MIC aligns with the MIC values reported from previous studies against *K. pneumoniae* [3]. The results of the time-kill assays demonstrate that the three tested antibiotics, CIP, GEN, and CEF, exhibit bactericidal activity against the tested strain of *K. pneumoniae* ATCC 13883 (Figure 2). In this experiment, we observed that GEN demonstrated enhanced activity against *K. pneumoniae* planktonic cells (Figure 2). From the results of this study, it is clear that complete killing of all *K. pneumoniae* was observed after 18 h of applying GEN (Figure 2). The antibiotics CIP and CEF achieved approximately 4.8 Log10 and 4 Log10 reductions, respectively, within 18 h (Figure 2).

Combining PDT and antibiotics does not always enhance efficacy, as the interaction depends on the therapy design and the bacterial strain. PDT has the potential to boost the antibacterial effectiveness of antibiotics primarily by facilitating the accumulation of PSs and activating photodynamic reactions through light exposure. This process can lead to the destabilization of bacterial outer structures, enhancing the overall efficacy of antibiotic treatment [6]. This would enhance the interaction of antibiotics with bacterial membranes or cell walls and promote the internalization of those that inhibit protein or nucleic acid synthesis, increasing overall antibacterial effects [6]. Additionally, PDT may impair bacterial resistance by inactivating drug-modifying enzymes, damaging efflux pumps, or increasing the binding affinity of mutated drug targets, making bacteria more sensitive to antibiotics [12]. Our study found that all three tested antibiotics showed synergistic effects with MB against *K. pneumoniae* (Figure 3A), while a moderate effect was observed with PDZ only at higher concentrations (Figure 3B). The checkerboard assay demonstrated that the inclusion of MB-mediated PDT significantly reduces the necessary doses of antibiotics such as CIP, GEN, or CEF. The synergy between photosensitizers and antibiotics appears to depend on the specific PS tested and the mechanisms of action of the antibiotics. Interestingly, when antibiotics are combined with MB at lower doses—roughly two to five times below their standard levels—they show similar effectiveness to when used alone (Figure 3A). This highlights the potential for improving antibiotic effectiveness when used alongside MB. The differences in actions among the various antibiotics used in this study and their synergistic effects are as follows.

### 3.1. PDT/Bacterial Nucleic Acid Synthesis Inhibiting Antibiotic Combinations

Ciprofloxacin is a common antibiotic used to treat bacterial infections. It is effective for infections in the urinary tract, respiratory system, digestive system, and skin. It works by blocking important enzymes in bacteria that are needed for DNA replication. These enzymes are called topoisomerase IV and DNA gyrase [13]. It has been reported that as the concentration of antibiotic CIP increases, PDT with photosensitizer MB did not produce significantly greater bactericidal effects than CIP alone against *Mycobacterium keratitis* [14]. This was likely due to competition between MB and CIP for efflux pumps, as well as interference caused by free radicals produced by PDT in the efficacy of cocultured CIP. However, our study found that PDT with MB enhances the antimicrobial effect of CIP against *K. pneumoniae* (Figure 3A). Our results showed that 0.35 µg/mL of CIP alone reduced bacterial count by one log10 (Figure 2); however, when combined with 1 µg/mL of MB, it completely inhibited the growth of *K. pneumoniae* (Figure 3A). Our in vitro data demonstrated that MB increases *K. pneumoniae*’s sensitivity to CIP, likely by destabilizing the bacterial cell membrane [15]. Our study highlights that combining MB and CIP enables the use of lower doses of CIP for treating *K. pneumoniae* infections. This strategy effectively reduces the pressure on bacteria to become resistant, which can help prevent the emergence of resistant strains.

### 3.2. PDT/Bacterial Protein Synthesis Inhibiting Antibiotic Combinations

Antibiotics inhibit bacterial protein synthesis by binding to their ribosomes. For instance, aminoglycosides like gentamicin bind irreversibly to the 30S subunit of the ribosome, inhibiting growth or killing bacteria [16]. Previous studies have reported that the combination of GEN and aPDT demonstrates superior qualitative and quantitative antimicrobial effects against *S. aureus* [17]. Green light laser irradiation (532 nm) with GEN enhances antibacterial activity against *P. aeruginosa* compared to either treatment alone [18]. In our study, the absence of PDT results in the necessity for a higher concentration of GEN, with a light dose of zero. However, as the dosage of PDT utilizing MB is systematically increased, the required concentration of GEN correspondingly diminishes, as illustrated in Figure 3A. It can be hypothesized that the greater reduction in CFUs observed in samples exposed to MB + GEN (Figure 3A), compared to those treated with either MB or GEN alone (Figure 1 and Figure 2, respectively), is due to a synergistic effect of GEN and the photoactivation of MB, as reported previously [18]. Our research supports previous studies that demonstrate a synergistic bactericidal effect when combining GEN and MB-aPDT against planktonic cultures of *S. aureus* and *P. aeruginosa* [19]. The synergistic effect observed in this study with MB-PDT and GEN might result from their complementary actions: PDT damages cellular structures, affecting membrane integrity, while GEN disrupts protein synthesis at the ribosome [19]. This combined stress overwhelms the bacteria’s repair mechanisms, leading to a higher death rate than either treatment alone. However, further studies are required to confirm this hypothesis.

### 3.3. PDT/Bacterial Cell Wall Synthesis Inhibiting Antibiotic Combinations

Ceftriaxone (CEF), a third-generation β-lactam cephalosporin antibiotic, inhibits bacterial cell wall formation by binding to penicillin-binding proteins (PBPs), which halts peptidoglycan production and exerts bactericidal effects [20]. Our study found that MB enhances the CEF effect, possibly by disrupting the cell wall synthesis of *K. pneumoniae* (Figure 3A).

The sequence in which PDT and antibiotics are administered plays a crucial role in the antimicrobial effectiveness of their combination. Research on PDT combined with bacterial cell wall-targeting antibiotics, like ceftriaxone (CEF), indicates that administering PDT and the CEF simultaneously is more likely to enhance the killing efficiency compared to using these treatments sequentially [21]. However, opinions differ in studies involving PDT combined with bacterial protein synthesis inhibiting antibiotics such as GEN, where the sequential application of the two drugs does not seem to be significantly less effective than concurrent treatment. For PDT combined with bacterial nucleic acid synthesis-inhibiting antibiotics such as CIP, the results are contradictory. For example, one report indicates that applying PDT before antibiotics results in 4 logs greater bacterial reduction than when antibiotics are applied before PDT, which barely reduces *E. coli* cells [22]. Conversely, applying PDT after the antibiotic resulted in 2 logs less bacterial reduction compared to when antibiotics preceded PDT, which achieved a 6-log reduction [23]. Our study shows that simultaneous application of PDT and an antibiotic produces a synergistic effect against *K. pneumoniae*. Research demonstrates that increasing the energy density of PDT significantly reduces the necessary concentration of antibiotics. This correlation is exemplified in Figure 4, which illustrates the decrease in antibiotic dosage required when supplemented with photodynamic treatment. It is important to note that unabsorbed PSs molecules have the potential to absorb activating light photons, which can distance them from the bacteria-bound PS. This phenomenon should be taken into account as a limitation of this study [24].

## 4. Materials and Methods

The overall experimental setup involves culturing microorganisms and sequentially applying the photosensitizer, light, and antibiotics, with final observations made after a certain period.

### 4.1. Growth Media

All media and dilutions were prepared following clinical antimicrobial susceptibility testing guidelines. Cation-adjusted Mueller-Hinton broth (CAMHB) and Mueller-Hinton agar (MHA) were purchased from Hardy Diagnostics (Santa Maria, CA, USA). Brain heart infusion broth (BHIB) and brain heart infusion agar (BHIA) were purchased from Millipore (Darmstadt, Germany) and prepared according to the manufacturer’s instructions.

### 4.2. Bacterial Strain and Culture Conditions

The microorganism used in this study is *Klebsiella pneumoniae* subsp. *pneumoniae*, obtained from the American Type Culture Collection (ATCC^®^ 13883™). It is grown at 37 °C for 24 h in Brain Heart Infusion (BHI; Millipore, Darmstadt, Germany) broth, and aliquots are frozen in BHI-glycerol at −80 °C until use. A pre-inoculum solution was prepared by mixing 1 mL of a cryotube bacteria sample with 1 mL of BHI (pH 7.4), then incubated at 37 °C for 18 h in a 5% CO_2_ atmosphere on a shaker incubator at 200 rpm (MaxQ 6000, Thermo Fisher Scientific, Waltham, MA, USA). Subsequently, the cells were collected, centrifuged for 5 min at 8000 rpm, and resuspended in phosphate-buffered saline (PBS). The optical density of the *K. pneumoniae* suspensions was adjusted to 0.2–0.3 at 600 nm (OD 600, Agilent Cary 60 UV-Vis, Agilent Technologies Inc., Santa Clara, CA, USA), corresponding to approximately 10^7^–10^8^ colony-forming units per milliliter (CFU/mL).

### 4.3. Antibiotic Preparation and Treatment

Stock solutions of Ciprofloxacin (CIP, purchased from AmBeed, cat# A208061), Gentamicin (GEN, Gentamicin sulfate, Thermo Fisher Scientific Inc., cat# 455310010), and Ceftriaxone (CEF, ceftriaxone disodium salt hemi heptahydrate, Thermo Fisher Scientific Inc., cat# 455420010) powder were dissolved in sterile distilled water and homogenized by vortex. The stock solution was then diluted in sterile PBS to the desired concentration following clinical antimicrobial susceptibility testing guidelines. The same protocol was applied to all three antibiotics—CIP, GEN, and CEF. The concentrations of each antibiotic used in combination with PSs are presented in Table 2.

### 4.4. Photosensitizer and Light Source

The photosensitizer methylene blue (MB) was obtained from Sigma-Aldrich (USP Reference Standard, cat# 1428008). A stock solution of MB (50 µg/mL) was prepared in sterile water and stored at 4 °C in the dark. Photodithazine^®^ (PDZ) is a chlorine e-6 supplied through the collaborative efforts of the University of São Paulo—Texas A&M University. The stock solutions of PDZ were prepared at concentrations of 400 µg/mL in PBS, then diluted in distilled water to reach the desired concentrations. PS solutions were prepared in microtubes wrapped in aluminum foil to prevent exposure to light during the experiment. The concentrations of each photosensitizer used, alone and in combination with antibiotics, are presented in Table 3.

**Figure 5:**
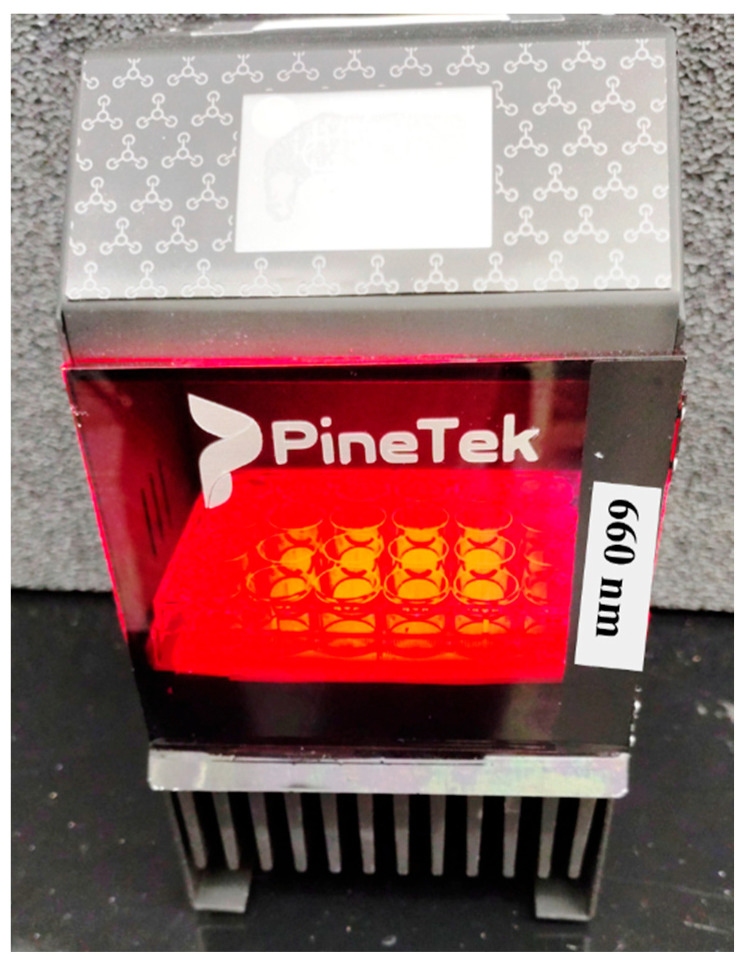
Diagram of the experimental irradiation setup used for photodynamic therapy (PDT). Irradiation was performed with a PineTek Biotable^®^—PINETEK LLC, College Station, TX, USA, a red light-emitting diode (LED)-based device that consists of 24 emitting centers with a wavelength of 660 nm. LED arrays were arranged so that each LED array was placed under a well from the well plate, providing the same average irradiation. The RSD values of irradiance obtained with this device were 75 mW/cm^2^. Variation from well to well remains at about 10%. The configuration allows for consistent and homogeneous light exposure, ensuring reproducibility of the photoinactivation experiments. The light dose applied was 15 J/cm^2^ at an exposure time of 3 min 20 s. In opposite to illuminating a single well each time, the device is more precise and reproducible.

The light source device for photodynamic therapy was obtained from the PineTek (PINETEK LLC, 511 University Dr., College Station, TX, USA) and built by the Technical Support laboratory from the Physics Institute of São Carlos (USP/SP/Brazil). The equipment is composed of a plate holder composed of 24 LEDs (4 × 6) with an irradiance of 75 mW/cm^2^, emitting homogeneously on the surface of the plates and operating at 660 nm. The distance between the end of the LED light guide and the sample surface was 80 mm. The device can be intensity and time controlled to provide the intended dose delivery. Variation from well to well stays at the 10% level. The overall view of the used device is represented in Figure 5.

### 4.5. Photodynamic Inactivation Procedure

The PDT procedure was followed by our recent publication [25]. In this study, we established three control groups to assess the effects of PDT: the general control group, which consisted of bacteria; the dark control group, which included bacteria combined with a PS; and the light control group, which comprised bacteria treated with a PS and light exposure. Additionally, we incorporated a treatment group that received the full PDT regimen, consisting of bacteria, a PS, and light. For the PDT and dark groups, bacteria were incubated with different doses of PSs for 20 min. Afterward, the light and PDT groups were exposed to a light dose of about 15 J/cm^2^ using a 660 nm LED device (Biotable, PINETEK LLC, College Station, TX, USA, Figure 5). The samples were then plated onto Petri dishes to count the number of surviving colony-forming units per milliliter (CFU/mL). The light dose was calculated with the equation *D = I × t*, where “D” is the light dose, “I” is the intensity of the irradiation device, and “t” is the irradiation time.

### 4.6. Determination of Minimum Inhibitory Concentration (MICs) of Antibiotics

The broth microdilution method was implemented to assess the MIC in line with the guidelines set forth by the Clinical and Laboratory Standards Institute (CLSI). The final inoculum in each well was approximately 1 × 10^5^ CFU/mL. Antibiotics—Ciprofloxacin (CIP), Gentamicin (GEN), and Ceftriaxone (CEF)—were distributed in 96-well plates through sequential dilution. The volume of PBS in each well was 90 μL, and each well was inoculated with 10 µL of logarithmic-phase *K. pneumoniae* bacterial inoculum, except for sterility control and growth control wells. A positive control for bacterial growth and a negative control of the medium were included, and the plate was incubated at 37 °C for 18 h. Thirty microliters of resazurin solution (0.015%) were added and incubated for 4 h at 37 °C. MIC was defined as the lowest concentration of an antibiotic that inhibits visible growth of the bacterial strains by converting resazurin into resorufin, which can be seen with the naked eye. All experiments were conducted in triplicate on different days.

### 4.7. Antibacterial Activity Assay

The antibacterial activity is assessed by colony-forming units (CFU). A time-killing assay was conducted to assess the effects of the antibiotics CIP, GEN, and CEF on *K. pneumoniae* by measuring the reduction in CFU per mL over a period of 18 h. The strains were grown in MHB starting with an inoculum of 10^6^ CFU/mL. The inoculum in MHB was measured to generate time-killing curves for each tested antibiotic alone on Petri dishes. MHB without antibiotics served as a control. Petri dishes were incubated at 37 °C for 18 h, then colonies were counted. All experiments were in triplicate.

### 4.8. Combination Effect of Photosensitizers and Antibiotics

**Figure 6:**
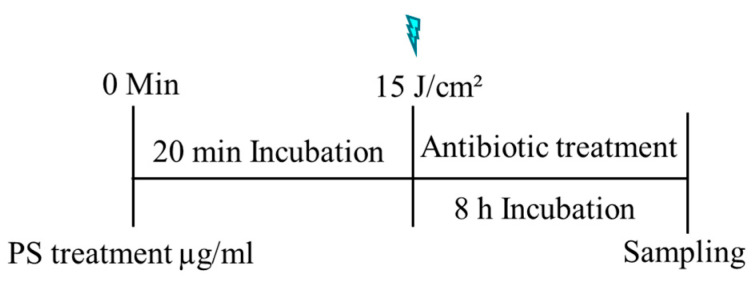
The schematic presentation of the combination effect of photosensitizers and antibiotics. At the beginning, the bacterial samples are treated with different doses of PS and incubated for 20 min in the dark at 37 °C and irradiated with a light dose of 15 J/cm^2^, except the control samples. Then the samples are incubated with antibiotics for 8 h at 37 °C. The samples were performed in triplicate and analyzed.

A checkerboard assay (CKB) was performed following our previously described method [26], to determine additivity or synergy between photosensitizers (MB or PDZ) and antibiotics (CIP, GEN, and CEF). The rows of 96-well plates were filled with *K. pneumoniae* bacterial suspension combined with various final PS concentrations of MB (0.2, 0.4, 0.6, 0.8 µg/mL), or PDZ (25, 50, 100 µg/mL). The prepared plates were incubated for 20 min at 37 °C in the dark, and then the samples were subjected to irradiation with a light dose of 15 J/cm^2^. The control group consisted of a *K. pneumoniae* bacterial suspension administered with either MB or PDZ but not treated with light. Then, 200 μL of different final antibiotic concentrations of CIP (0.00005, 0.0001, 0.005, 0.025, 0.35 µg/mL), or GEN (0.03, 0.08, 0.13, 0.25, 0.5 µg/mL), or CEF (0.03, 0.08, 0.17, 0.35, 0.7 µg/mL), solutions were added to each well, and the plates were incubated for a further 8 h at 37 °C in the dark. Consecutively, each well was diluted by serial dilution, plated in BHI agar, and incubated for 18 h at 37 °C to determine the CFU/mL. In Figure 6, the overall time sequence of the methodology applied for the experiment is illustrated. The interactions between the combined PSs and antibiotics can be derived from the fractional inhibitory concentration index (FICI), which is expressed as FIC_I_ = FIC_P_ + FIC_AB_ = FIC_PAB_/FIC_AB_ + FIC_ABP_/FIC_P_. The FIC_P_ and FIC_AB_ are the concentrations of PSs and antibiotics, respectively, when acting alone, and FIC_PAB_ and FIC_ABP_ are the concentrations of PSs and antibiotics, respectively, when acting in combinations. If FIC_I_ ≤ 0.5—synergy; 0.5 < FIC_I_ ≤ 1.0—additive; 1.0 < FIC_I_ ≤ 4.0—indifferent; 4.0 < FIC_I_—antagonism.

### 4.9. Statistical Analysis

All experiments were performed in triplicate on three independent occasions, and the results are presented as averages. All data points were expressed as the mean ± standard deviation (SD). The data were tested for normality using the Shapiro–Wilk test. Two-group comparisons were performed with Student’s *t*-test, and one-way ANOVA followed by Tukey’s test was used to compare the CFU/mL counts across different experimental groups. When appropriate, post hoc comparisons were conducted with the Tamhane test. A *p*-value of less than 0.05 was considered statistically significant, indicating a meaningful difference between treatment groups. All analyses were performed using Origin^®^ software version 2024b, with a license granted by Texas A&M University.

## 5. Conclusions

In conclusion, this study showed that when irradiated with 15 J/cm^2^ of 660 nm light, methylene blue (MB) was a more effective growth inhibitor of K. pneumoniae compared to Photodithazine (PDZ), and the light energy doses significantly affected the growth of *K. pneumoniae*. The tested antibiotics, ciprofloxacin (CIP), gentamicin (GEN), and ceftriaxone (CEF), reduced bacterial growth and effectively killed K. pneumoniae. Additionally, applying PDT with MB and the antibiotics CIP, GEN, and CEF sequentially produced a synergistic effect against *K. pneumoniae*. This study highlights the promising potential of pre-treating bacterial cells with a photosensitizer (PS) and light to increase their susceptibility to antibiotics. This approach could offer an innovative alternative for treating local infections caused by antibiotic-resistant bacteria, including *K. pneumoniae*. An important insight from our research is that combining PDT with an antibiotic may significantly reduce the required dosage of the antibiotic to achieve effective bacterial elimination. The observed synergistic effect depends on the specific combination of PS and antibiotics used and may also be influenced by the energy delivered during treatment; however, this aspect was not addressed in the current study. We plan to conduct a mathematical analysis of the observed behavior of the combination and propose universal constants related to bacteria, antibiotics, and PS to assist future PDT and antibiotic combination therapies.

## Figures and Tables

**Table 1:** Minimal inhibitory concentrations of the tested antibiotics CIP, GEN, and CEF.

Bacteria	Antibiotic	MIC (µg/mL)
*K. pneumoniae*	Ciprofloxacin	0.1875
	Gentamicin	1.5
	Ceftriaxone	2.125

**Table 2:** The concentrations of each antibiotic used in combination with PSs.

Antibiotic	Concentrations Used (µg/mL)
Ciprofloxacin (CIP)	0.00005, 0.0001, 0.005, 0.025, 0.35
Gentamicin (GEN)	0.03, 0.08, 0.13, 0.25, 0.5
Ceftriaxone (CEF)	0.03, 0.08, 0.17, 0.35, 0.7

**Table 3:** The concentrations of each photosensitizer used, alone and in combination with antibiotics.

Photosensitizer	Concentrations Used Alone (µg/mL)	Concentrations Used in Combination with Antibiotics (µg/mL)
Methylene blue (MB)	0.25, 0.5, 0.75, 1.0	0.2, 0.4, 0.6, 0.8
Photodithazine (PDZ)	25, 50, 100	25, 50, 100

## Data Availability

The original contributions presented in this study are included in the article. Further inquiries can be directed to the corresponding author.
